# Supplementary material for: Targeting prostate cancer by new bispecific monocyte engager directed to prostate-specific membrane antigen
Source: PLoS One. 2025 Mar 17;20(3):e0307353. doi: 10.1371/journal.pone.0307353 (PMC11913275; doi:10.1371/journal.pone.0307353)
Supplement: S1 Table — (PDF) [file pone.0307353.s001.pdf]

S1 Table. List of primers used for cloning of 5D3/CP33 variants and chimeric 5D3 molecule.

Primers	Sequence
5D3-CP33	**Forward** - AAAAAGATCTGAAGTTCAGCTGCAGCAGAG
	**Reverse** - TTTTCTCGAGTCATTATTTTTCGAACTGC
CP33-5D3	**Forward** - AAAAAGATCTGTTAATAGCTGTCTGCTGC
	**Reverse** TTTTCTCGAGTCATTATTTTTCGAACTGC
pVITRO A	**Forward** - CGTACGGTGGCGGCGCCATCTGTCTTCATCTTCCCGCCAT
	**Reverse** - GGAGTGCGCGCCTGTGGCGGCCGCCACCAAGAAGAGGATC
pVITRO B	**Forward** - AGTCTCCTCAGCTAGCACCAAGGGCCCATC
	**Reverse** - TCTGGATGTCACCGCGGCTAGCTGGAAC
5D3 HV	**Forward** - CCGCCACAGGCGCGCACTCCGAGGTTCAGCTTCAGCAGTCTGGACCTG
	**Reverse** - TGGTGCTAGCTGAGGAGACTGTGAGAGTG
5D3 LV	**Forward** - TAGCCGCGGTGACATCCAGATGACCCAG
	**Reverse** - GATGGCGCCGCCACCGTACGAGCCCGTTTTATTTCCACCT
