# Supplementary material for: Targeting prostate cancer by new bispecific monocyte engager directed to prostate-specific membrane antigen
Source: PLoS One. 2025 Mar 17;20(3):e0307353. doi: 10.1371/journal.pone.0307353 (PMC11913275; doi:10.1371/journal.pone.0307353)
Supplement: S1 Fig — (PDF) [file pone.0307353.s002.pdf]

S1 Figure

**5D3-CP33**

MKLCILLAVVAFVGLSLGRSEVQLQQSGPELVKPGASVKISCKTSGYAFNTSWMNWVKQRPGQGLEWIGRIYPGDGDTNYNG
KFKGKATLTADKSSSTAHMHLSSLTSVDSAVYFCARGEWYLYYFDYWGQGTTLTVSSGGSGGGGSGGGGSGGGGSDIQMTQT
TSSLSASLGDRVTISCSASQGINNFLTWYQQKPDGTLKLLIYYTSSLHSGVPSRFSGSGSGTDYSLTIRNLEPEDIATYYCQ
QYSNLPFTFGGGTKVEIKRAGTGGGSGGGSGGGSGVNSCLLLPNLLGCGDDGTSAWSHPQFEK

S-S

**CP33-5D3**

S-S

MKLCILLAVVAFVGLSLGRSVNSCLLLPNLLGCGDDGTGGGSGGGSGGGSGEVQLQQSGPELVKPGASVKISCKTSGYAFNT
SWMNWVKQRPGQGLEWIGRIYPGDGDTNYNGKFKGKATLTADKSSSTAHMHLSSLTSVDSAVYFCARGEWYLYYFDYWGQGT
TLTVSSGGSGGGGSGGGGSGGGGSDIQMTQTTSSLSASLGDRVTISCSASQGINNFLTWYQQKPDGTLKLLIYYTSSLHSGV
PSRFSGSGSGTDYSLTIRNLEPEDIATYYCQQYSNLPFTFGGGTKVEIKRASAWSHPQFEK

S1 Figure: Scheme of 5D3 monocyte engager variants. The N-terminal BiP secretion signal sequence and affinity SA-Strep II tag are colored grey and purple, respectively.  Antibody variable domains of 5D3 and CP33 are colored green and orange, respectively. The disulphide linkage between 2-cysteines in CP33 peptide is marked in black.
