# Supplementary material for: Targeting prostate cancer by new bispecific monocyte engager directed to prostate-specific membrane antigen
Source: PLoS One. 2025 Mar 17;20(3):e0307353. doi: 10.1371/journal.pone.0307353 (PMC11913275; doi:10.1371/journal.pone.0307353)
Supplement: S2 Fig — (PDF) [file pone.0307353.s003.pdf]

S2 Figure

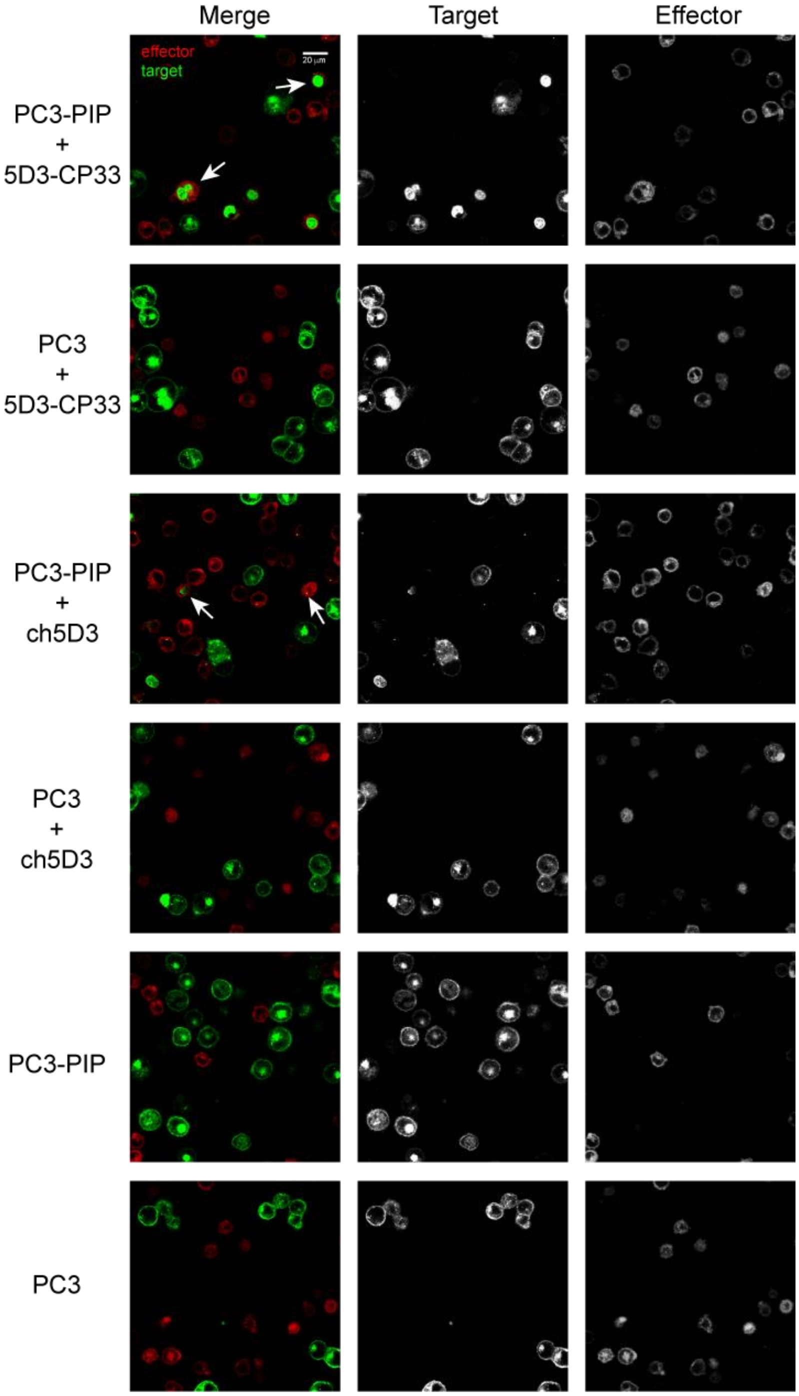

S2 Figure: Specific phagocytosis of PSMA-positive cells. Confocal microscopy images show double positive objects that correspond to target cells (green) engulfed by U937 monocytes (red), representative objects marked by arrows. PC3/PC3-PIP cells and U937 cells were labeled by DiO and DiD dye, respectively, and then co-cultivated in the presence of 111 nM 5D3-CP33. Process of engulfement was monitored on alive cells by confocal microscope.
