# Supplementary material for: Targeting prostate cancer by new bispecific monocyte engager directed to prostate-specific membrane antigen
Source: PLoS One. 2025 Mar 17;20(3):e0307353. doi: 10.1371/journal.pone.0307353 (PMC11913275; doi:10.1371/journal.pone.0307353)
Supplement: S1 Raw Images — (PDF) [file pone.0307353.s004.pdf]

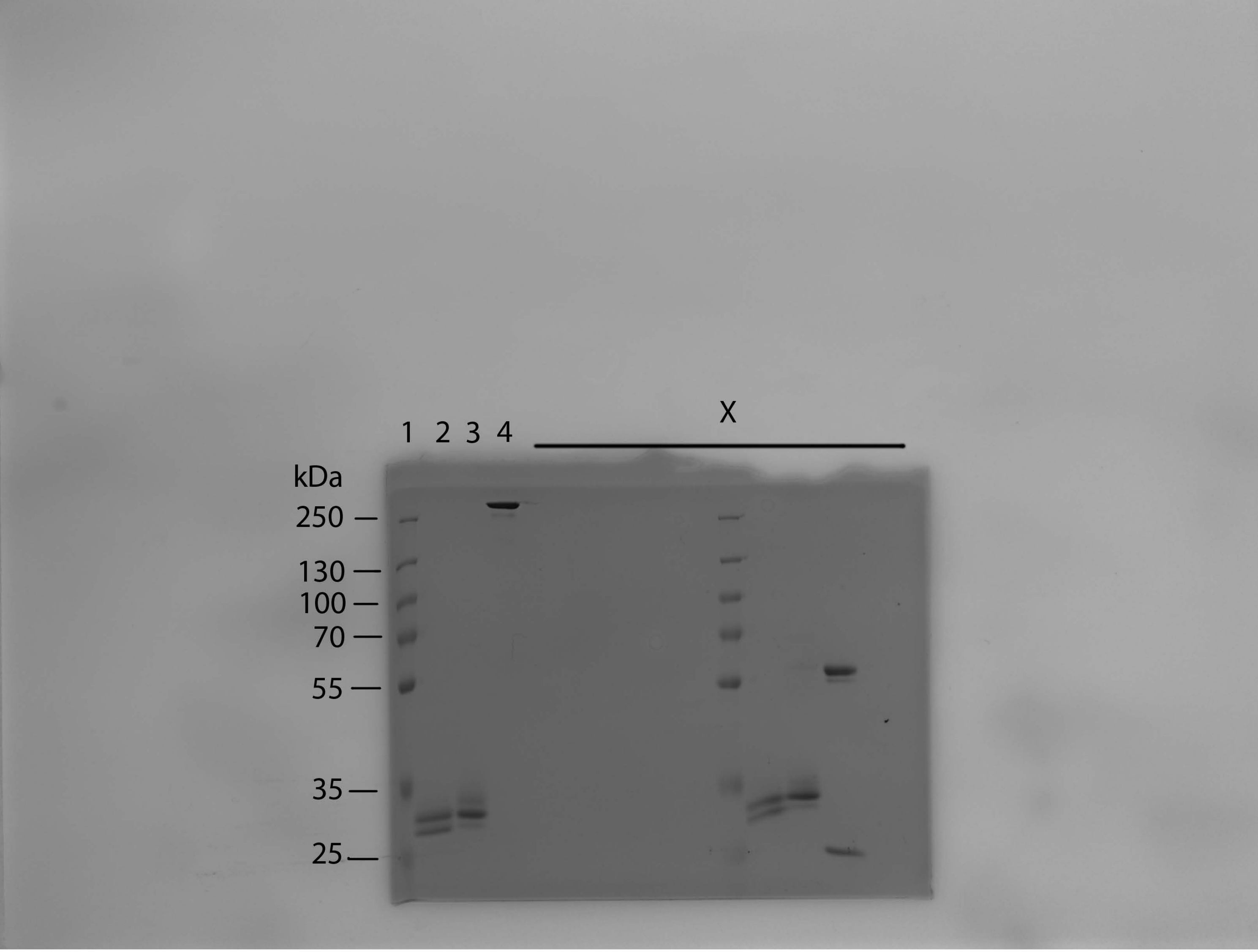

CBB-stained gel imaged by CCD camera - raw gel image corresponding to Fig 1D.

lane 1 - marker

lane 2 - 5D3-CP33

lane 3 - CP33-5D3

lane 4 - chimeric 5D3 IgG1
