# Supplementary material for: Targeting prostate cancer by new bispecific monocyte engager directed to prostate-specific membrane antigen
Source: PLoS One. 2025 Mar 17;20(3):e0307353. doi: 10.1371/journal.pone.0307353 (PMC11913275; doi:10.1371/journal.pone.0307353)
Supplement: Attachment — Submitted filename: Response to Reviewers.pdf [file pone.0307353.s005.pdf]

Vestec, January 24, 2025

Review Comments to the Author

**Reviewer #1**: The paper aims to develop and functional characterize a bispecific monocyte engager capable of simultaneously targeting PSMA-positive cancer cells and the Fc gamma receptor I (FcγRI/CD64 ) receptor present on the monocyte/macrophage surface. As far as this paper is in my field of expertise (except technical aspects), the manuscript is well written, clear and of interest to readers, thanks to the increasing interest that PSMA has widely spread in recent years. Due to my limited experience in chemistry and radiochemistry, I cannot judge the technical parts.

**Reviewer #2**: The paper needs importante amendments
Demonstrate engagement of human macrophage
U937 alone Is not convincing

**Authors' answer**: Based on reviewer's suggestion, effect and specificity of 5D3-CP33 bispecific engager was evaluated on human macrophages differentiated from monocytes (isolated from human PBMCs). Data was inserted as panels D,E in Figure 4. The presence of 5D3-CP33 enhances specific phagocytosis of PSMA-positive but not PSMA-negative cells.

In this study, the U937 cell line was employed as a well-established and standardized model routinely used to evaluate phagocytosis. We believe that results related to isolated human macrophages that were added to revised manuscript strengthen main findings of the study.

**Reviewer #2**: Perform functional experiments at different E/T ratios

**Authors' answer**: We appreciate reviewer's comment. Numerous previously published studies show specific phagocytosis of tumor cells driven by linkage via recombinant/synthetic molecules (see papers specified by DOI: 10.1002/ange.201510866, 10.1021/acschembio.0c00112, 10.1002/cpch.88, 10.1021/acscentsci.3c01052, 10.1016/j.biomaterials.2020.120601, 10.1080/19420862.2020.1857100, 10.4049/jimmunol.1402891, 10.1016/j.jim.2007.04.009, 10.1091/mbc.E03-09-0668, 10.18632/oncotarget.18492). In these studies, that utilized monocyte cell lines as well as freshly isolated human monocytes, the E/T ratio varied from 4:1 to 1:1 with significant preference of ratio 1:1. Since our data show superior rate of phagocytosis at E/T ratio 1:1 (Figure 4) that represents standard ratio used preferentially in experimental studies, we conclude that increase in the amount of effector cells would not bring any significant benefit to our study.

Specific phagocytosis-supporting effect of 5D3-CP33 was observable even when target cells were present in excess over effectors represented by freshly isolated human monocytes differentiated and polarized into M1 type macrophages (E/T ratio was approximately 0.8:1), see Figure 4E.

**Reviewer #2**: A construct control lacking PSMA Binding is needed

**Authors' answer**: We agree with reviewer that implementation of proper controls is critical for the study. The specific binding of both parts of the bispecific engager was evaluated in detail and verified on effector and target cells using flow cytometry (see data in Figure 2 of our study). To show specific effect of 5D3-CP33, we implemented in our study two types of negative control: 1) a mixture of cells with the absence of the bispecific molecule, and 2) PSMA-negative target cells highly similar to

PSMA-positive target (PSMA-positive cells represent original cell line overexpressing recombinant PSMA). First negative control shows potential non-specific phagocytosis, whereas second negative control simulates the absence of PSMA binding and is equal to the construct lacking PSMA binding part as suggested by reviewer. The results clearly indicate that PSMA-negative cell line is not attracted by the macrophages, even though 5D3-CP33 engager is present in concentration as high as 1 µM in cell mixture. Both negative controls prove specific binding and stimulatory effect of 5D3-CP33 in phagocytosis of PSMA-positive cells. Additionally, our study includes IgG1 molecule with standard Fc part that serves as a positive control (see Figure 4 of our study), therefore readers could assess directly the efficiency of CP33 part of bispecific engager in binding and activation of macrophages.
